# Literary Notes

**Published:** 1906-10

**Authors:** 


### LITERARY NOTES.

The International Journal of Therapy has lately presented its appearance in the journalistic field. It is well edited and its make-up is attractive. It is limited to the topic of therapeutics as indicated by its title. It is edited by Dr. Otto Juettner, of Cincinnati. The subscription price is one dollar a year.

---

The International Medical Review, to be published in Berlin and edited by Dr. Hugo Neuman, is announced to appear at an early day. It will be printed in English, German, French, Italian, Spanish, and Portuguese, while a Russian edition is projected. It is intended to be a journal for medical practitioners, hence the important questions of therapy and diagnosis will receive greatest prominence. The first number will be looked forward to with great interest, and we bespeak for this new enterprise the success which the magnitude of the undertaking so well deserves.

